# Elucidating hepatocellular carcinoma progression: a novel prognostic miRNA–mRNA network and signature analysis

**DOI:** 10.1038/s41598-024-55806-y

**Published:** 2024-02-29

**Authors:** Fei Wang, Xichun Kang, Yaoqi Li, Jianhua Lu, Xiling Liu, Huimin Yan

**Affiliations:** Clinical Research Center, Shijiazhuang Fifth Hospital, Shijiazhuang, Hebei China

**Keywords:** Hepatocellular carcinoma, miRNA, Prognosis, miRNA–mRNA network, Cancer models, Cancer, Molecular medicine, Risk factors

## Abstract

There is increasing evidence that miRNAs play an important role in the prognosis of HCC. There is currently a lack of acknowledged models that accurately predict patient prognosis. The aim of this study is to create a miRNA-based model to precisely forecast a patient’s prognosis and a miRNA–mRNA network to investigate the function of a targeted mRNA. TCGA miRNA dataset and survival data of HCC patients were downloaded for differential analysis. The outcomes of variance analysis were subjected to univariate and multivariate Cox regression analyses and LASSO analysis. We constructed and visualized prognosis-related models and subsequently used violin plots to probe the function of miRNAs in tumor cells. We predicted the target mRNAs added those to the String database, built PPI protein interaction networks, and screened those mRNA using Cytoscape. The hub mRNA was subjected to GO and KEGG analysis to determine its biological role. Six of them were associated with prognosis: hsa-miR-139-3p, hsa-miR-139-5p, hsa-miR-101-3p, hsa-miR-30d-5p, hsa-miR-5003-3p, and hsa-miR-6844. The prognostic model was highly predictive and consistently performs, with the C index exceeding 0.7 after 1, 3, and 5 years. The model estimated significant differences in the Kaplan–Meier plotter and the model could predict patient prognosis independently of clinical indicators. A relatively stable miRNA prognostic model for HCC patients was constructed, and the model was highly accurate in predicting patients with good stability over 5 years. The miRNA–mRNA network was constructed to explore the function of mRNA.

## Introduction

Liver cancer is the sixth most common type of cancer that poses a serious threat to human health^1^. There have been 905,677 new cases reported worldwide. Cancer-related mortality will rank fourth in 2020, with 830,180 deaths^2^. Of all primary liver cancer, about 75–80% are hepatocellular carcinoma (HCC)^3,4^. The prognosis for patients with HCC after a diagnosis is still dismal, despite increased research into early diagnosis and ways to improve that prognosis^5^. It is reported that the 5-year survival rate after radical surgical surgery for HCC patients remains less than 50%^6^. This suggests that despite radical resection, the prognosis for HCC is unsatisfactory. At present, the published prognostic prediction models are difficult to satisfy and struggle with high certainty issues^7^. Therefore, it is urgent to discover potential biomarkers and therapeutic targets, construct more accurate and clinically accessible genetic information prediction models to predict patient prognosis and achieve precise treatment.

In recent years, microRNAs (miRNAs) have become a popular area of oncology research. MiRNAs are a group of endogenous single-stranded non-coding RNA molecules containing approximately 19–25 nucleotides^8,9^. They have an effect on gene expression at the post-transcriptional level^10^. An increasing number of studies have shown that miRNAs play a role in promoting tumor development or inhibiting tumor progression^11–13^. Meanwhile, many studies have also identified the role of miRNAs in tumor prognosis^14^. It has been confirmed that miRNA has a good predictive ability for patients’ prognosis in cancers such as esophageal cancer^15^, colorectal cancer^16^ and breast cancer^17^. Studies have found that miRNAs are aberrantly expressed in HCC and are involved in the growth, development and metastasis of HCC by acting as oncogenes or tumor suppressors^18^. However, there is no authoritative model to predict the prognosis of patients and achieve treatment in the prognosis of hepatocellular carcinoma. Therefore, a miRNA-based prognostic model for HCC patients is urgently needed to accurately predict the prognosis of patients and achieve targeted therapy to prolong their overall survival.

In this study, we identified differentially expressed miRNAs (DEmiRNAs), constructed a prognostic model to predict patient prognosis, and investigated the roles of prognosis-related miRNAs and their associated mRNAs. This study will help to understand the role of miRNAs and achieve accurate prognosis prediction in HCC patients.

## Materials and methods

### Data source

miRNA expression profiles and clinical information were downloaded from the cancer genome database (TCGA), which includes 372 HCC samples and 50 adjacent normal tissue samples as of February 13, 2022. The external validation dataset GSE227378 was obtained from the GEO database and included 32 HCC cases and 32 adjacent normal tissue samples. The data in the TCGA and GEO database are publicly available and open access, and this study follows the database access policy and publication guidelines.

### Screening of differentially expressed miRNAs between HCC tissues and normal tissues

The raw data were corrected, filtered, and normalized using the R package. A total of 2652 miRNAs were included after integration. Subsequently, differential analysis was performed using the edgeR package in the environment of R4.1.0. The miRNAs with │log_2_FC│ > 1 and *P* < 0.05 were identified as DEmiRNAs.

### Establishment of the gene-related prognostic model

Data with missing survival data and survival time less than 30 days were excluded. HCC patients were randomly divided into a training set and a validation set according to 7:3 using the caret package in the environment of R.

Univariate and multivariate Cox regression analyses and Lasso regression analysis was used to investigate the association between DEmiRNA expression levels in HCC tissues and the overall survival (OS) of patients in the training set. *P* < 0.05 was considered significant in the results of univariate and multivariate Cox regression analyses. Significant results were then placed into the Lasso-penalized Cox analysis for further screening. Lasso-penalized Cox analysis with penalty parameter tuning performed via tenfold cross-validation was established to further narrow the miRNAs in which we required selected miRNAs to appear over 900 times for a total of 1000 repetitions. The final result is thought to be related to miRNAs those affect patient survival. Finally, a total of six miRNAs were left based on the minimum criteria of coefficients. These miRNAs were reincorporated into the Cox regression model for fitting and constructing a prognostic model.

### Visualization and validation of prognostic model

A multivariate Cox regression model was used to assess patient survival at 1-, 3-, and 5- years in the training set. The results were subsequently presented more visually using nomogram. The receiver operating characteristic curves (ROC) and C-index were used to validate the discrimination of the model in the training set, validation set, total set, and external validation set GSE227378. Calibration curve was used to assess the accuracy, and decision curve analysis (DCA) was used to assess the clinical utility of the model at 1-, 3-, and 5- years.

The risk score for each HCC patient was the regression coefficient derived from a multifactorial Cox regression model multiplied by the miRNA expression level, and the optimal cutoff value was determined using R. The optimal cut-off values of HCC patients with survival data in the training set, validation set, total set and external validation set GSE227378 were calculated in the environment of R. The predictive model was characterized by the linear combination of the expression levels of the six miRNAs weighted by their relative coefficient in the multivariate Cox regression. Risk score = (β1 × miRNA1 expression) + (β2 × miRNA2 expression) + … + (βn × miRNAn expression). The patients were divided into high-risk and low-risk groups according to the optimal cut-off values. The corresponding survival curves and survival state diagrams were plotted in each set according to the grouping using the survival package in R language. The difference in prognosis between the two groups of HCC patients was determined based on the graphs.

### Independence of the prognostic model from other clinical indicators

To determine whether the predictive ability of the model could be independent of other clinical indicators (age, gender, grade, stage, T) in HCC patients, univariate and multivariate Cox regression analyses were performed with other clinical indicators and the model as independent variables and the OS of patients as dependent variables.

### The role of prognosis-related miRNAs in tumor cell proliferation, invasion and metastasis

To explore the possible role of prognosis-related miRNAs in tumor development, we used R to plot the expression of these miRNAs in TNM stages of HCC patients as a violin graph. The mean values were compared to determine the tumor in expression and thus to speculate the role played by these miRNAs in tumor cell proliferation, invasion, and metastasis. Kruskal–Wallis test was used for T-stage, and Mann–Whitney U test was used for N-stage and M-stage. The test level *α* was taken as 0.05.

### Prediction of target mRNAs for each of the 6 miRNAs

The corresponding mRNAs for the six miRNAs were predicted using the miRDB, Targetscan, and miTarbase databases. The predicted results of these three databases were taken as the intersection, and the intersection results were displayed as the final results in the form of a Venn diagram.

### Screening of target mRNAs to derive hub mRNAs

The target mRNAs were imported into the STRING database to build a protein–protein interaction (PPI) network and observe the mRNA relationship. We used Cytoscape V3.7.1 to visually transform the results to investigate the link between target mRNAs. The results of the STRING database were imported into Cytoscape database. The CytoHubba in Cytoscape software was used to calculate the degree values of genes, and the top 15 genes with the highest degree values were selected as the hub genes. Subsequently, the relationship between hub mRNAs and mRNAs and up- and down-regulation in tumor tissues were linked by Sankey diagram.

### Functional enrichment analysis

To explore the function of hub mRNAs, Gene Ontology (GO) and Kyoto Encyclopedia of Genes and Genomes (KEGG) pathway^19–21^ enrichment analyses of hub mRNAs were analyzed using the R language clusterProfiler package, and the results were visualized by the SRplot platform.

## Results

### Differentially expressed miRNAs between HCC and normal tissues

**Figure 1:**
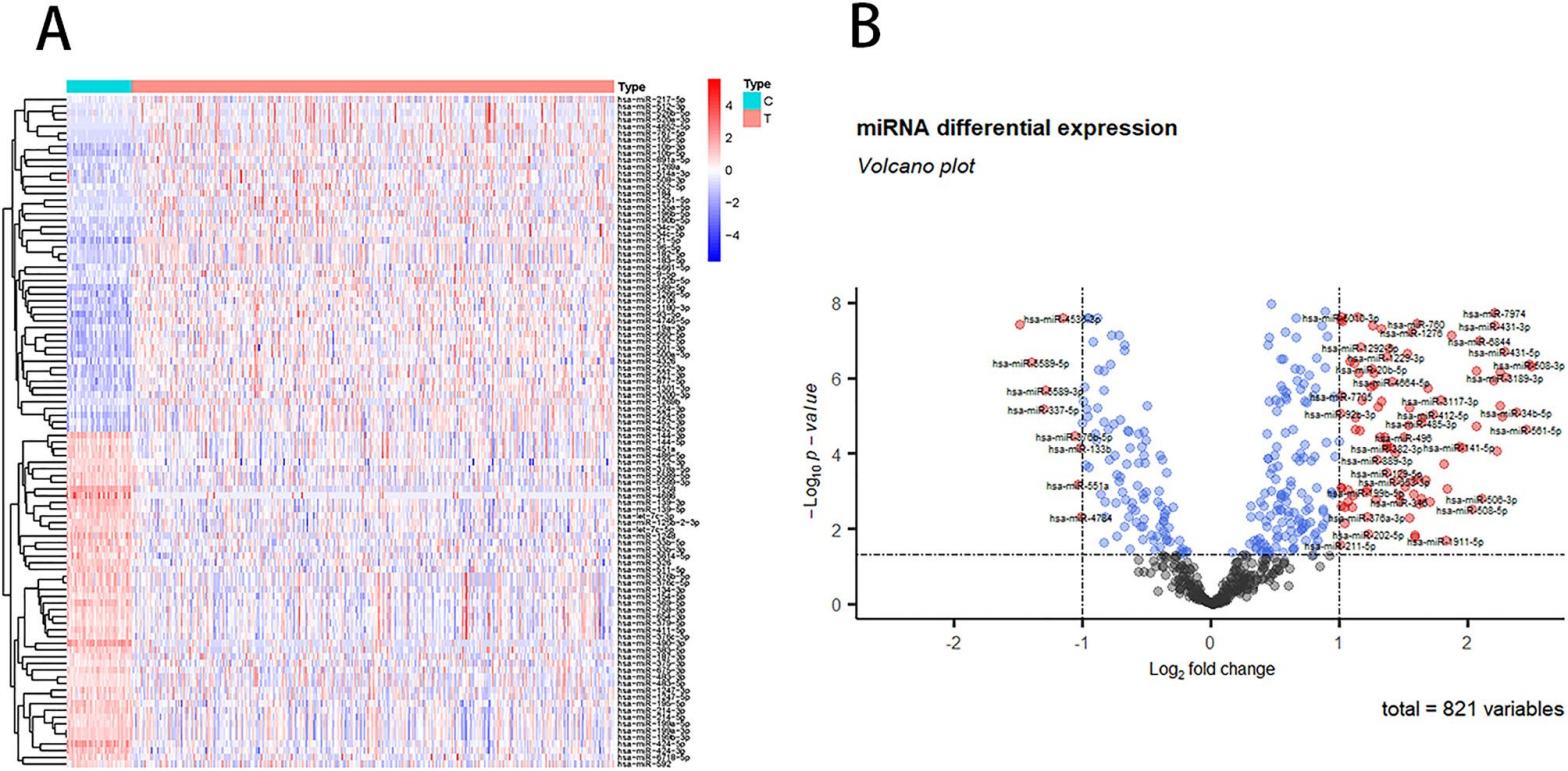
Heatmap (**A**) and volcano map (**B**) of differentially expressed miRNAs in HCC samples and normal tissue samples. In the volcano map, the vertical axis indicates differentially expressed miRNA. The horizontal axis indicates samples. Red represents high expression in HCC and blue represents low expression in HCC.

A total of 305 DEmiRNAs between HCC and normal tissues were identified, which containing 258 up-regulated miRNAs and 47 down-regulated miRNAs. The top 50 up-regulated DEmiRNAs and 47 down-regulated DEmiRNAs were shown in Fig. 1A. The distribution of the DEmiRNAs was shown in Fig. 1B.

### Identification of key miRNAs and construction of prognostic model

**Figure 2:**
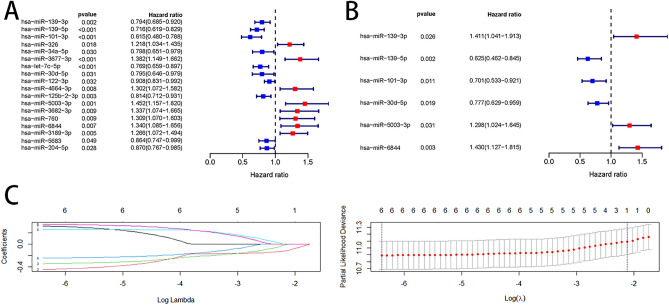
Differential miRNAs associated with prognosis of HCC patients. Univariate Cox analysis (**A**) and multivariate Cox analysis (**B**) of differential miRNAs, with red representing risk factors and blue representing protective factors. Lasso’s screening process for 6 miRNAs (**C**).

Univariate Cox regression analysis was performed on the DEmiRNAs in the training set. The results showed that 18 miRNAs were associated with OS of HCC patients (Fig. 2A). All significant results from the univariate Cox regression analysis were included in the multivariate Cox regression model for analysis. As a result, six miRNAs were identified (Fig. 2B).

Subsequently, the significant results from the multivariate Cox regression were included in the Lasso-penalized Cox for further screening. The results showed that six miRNAs, hsa-miR-139-3p, hsa-miR-139-5p, hsa-miR-101-3p, hsa-miR-30d-5p, hsa-miR-5003-3p, and hsa-miR-6844, were associated with the prognosis of HCC patients (Fig. 2C). The predictive model was characterized as follows: risk score = (0.3444 * expression level of has-miR-139-3p) + (− 0.4698* expression level of has-miR-139-5p) + (− 0.3556* expression level of has-miR-101-3p) + (− 0.2528 * expression level of has-miR-30d-5p) + (0.2608* expression level of has-miR-5003-3p) + (0.3576 * expression level of has-miR-6844).

### Construction of nomogram and validation of the model

**Figure 3:**
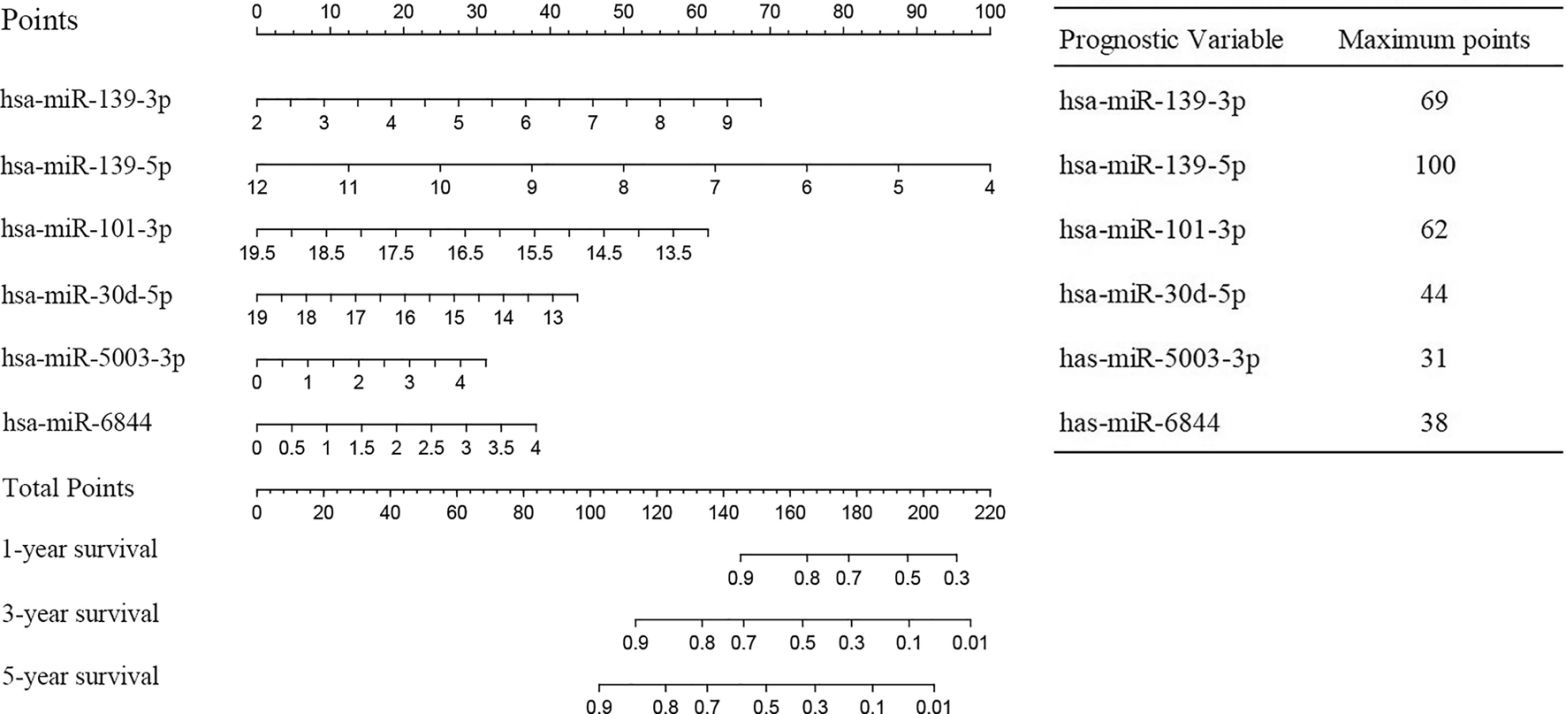
Nomogram predicting 1-, 3-, and 5-year survival rates for patients with HCC. The nomogram is applied by adding up the points identified on the points scale for each variable. The total points projected on the bottom scales indicate the probability of 1-, 3- and 5-year OS.

**Figure 4:**
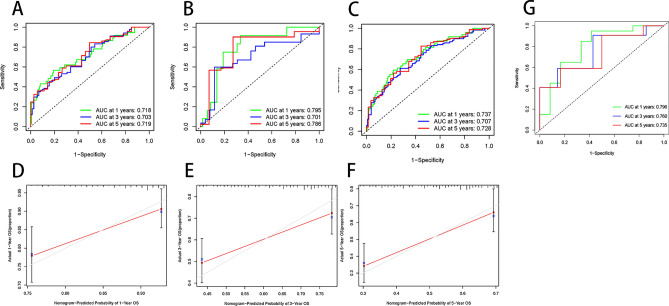
ROC curve and calibration curve of the model. ROC curves of the model in the training set (**A**), validation set (**B**), total set (**C**), and external validation set (**G**) with green representing 1 year, blue representing 3 years, and red representing 5 years. Calibration curves for 1 year (**D**), 3 years (**E**), and 5 years (**F**). The red line indicates the predicted situation and the gray line indicates the actual situation.

The six prognosis-related miRNAs were reincorporated into the Cox regression model for fitting, and the results were visualized by nomogram (Fig. 3). The predictive ability of the model was judged by the ROC curves. The results showed that all of AUC values 1-, 3-, and 5-year survival were more than 0.7 in the training set (Fig. 4A), test set (Fig. 4B), total set (Fig. 4C), and and external validation set GSE227378 (Fig. 4G) suggesting that the model had a better predictive performance.

**Figure 5:**
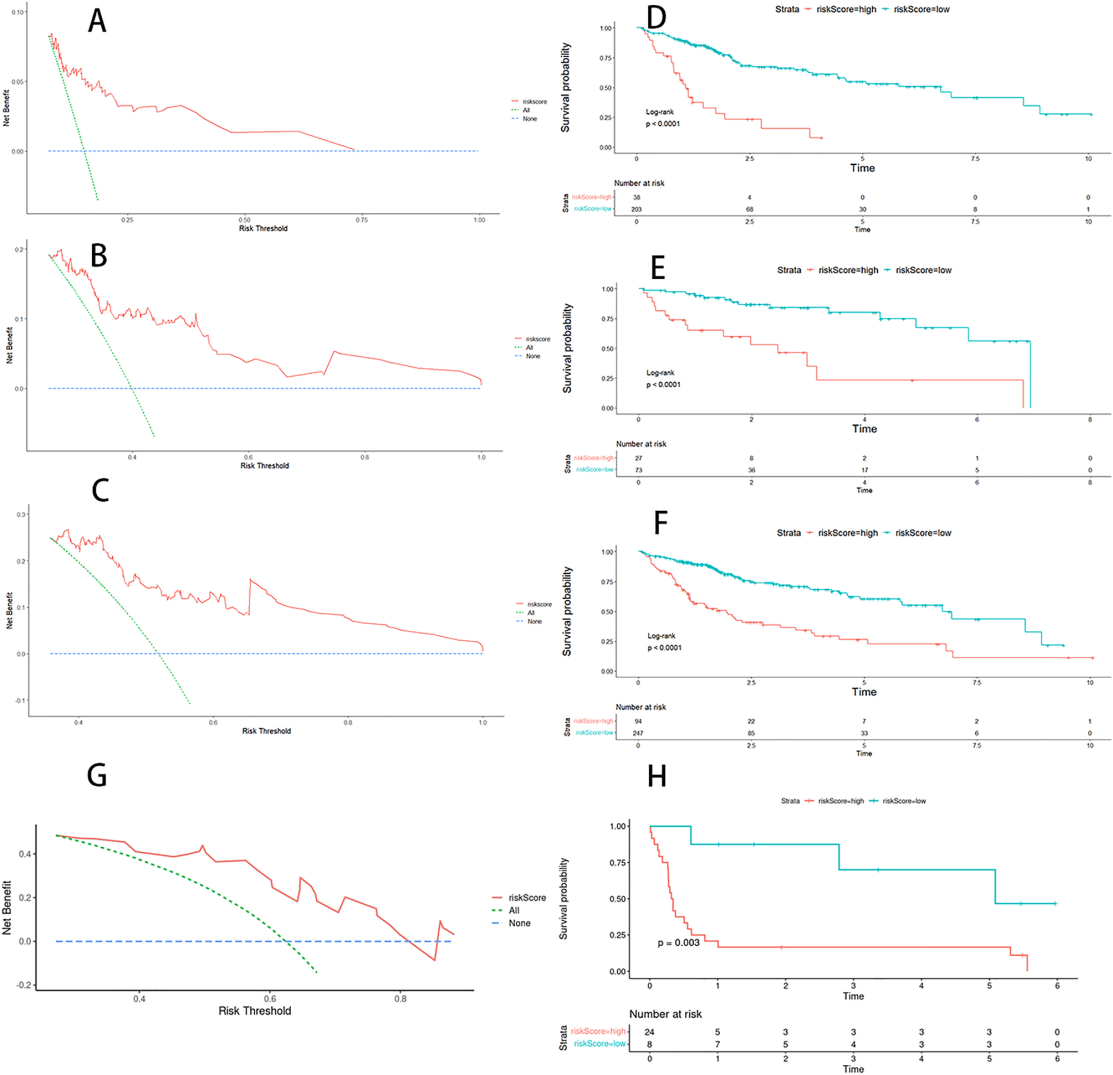
DCA curve and survival curve of the model. DCA curves for models 1 year (**A**, **G**), 3 years (**B**), and 5 years (**C**). KM survival curves plotted in the training set (**D**), test set (**E**), total set (**F**), and external validation set (**H**) using the optimal cut-off point for each grouping. Blue represents the low-risk group and red represents the high-risk group.

The accuracy of the model was assessed using calibration curves. The results showed that the nomogram performed well at 1-, 3-, and 5-year (Fig. 4D–F). The clinical usefulness of the model was assessed by the DCA curve. The results showed a high clinical benefit of the model in each set (Fig. 5A,B,C,G), which may help in patient counseling, decision making and follow-up.

**Figure 6:**
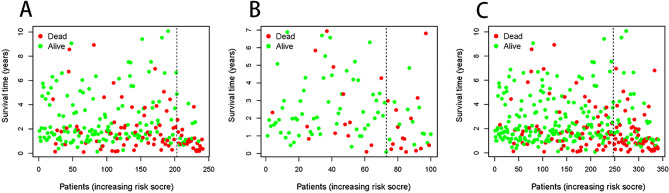
Survival state diagram by risk grouping. Survival status plots for the training set (**A**), test set (**B**), and total set (**C**). The horizontal coordinates indicate the risk score and the vertical coordinates indicate the survival time. The dashed line indicates the optimal cut-off point, red dots indicate that the outcome event has occurred, and green dots indicate that the outcome event has not occurred.

The survival curves between the two groups in each set were plotted by grouping the models by the optimal cut-off point (Fig. 5D,E,F,H), and the survival status (Fig. 6A–C, G) of the samples in each dataset was plotted by risk grouping. The results showed that in each set, the prognosis of the high-risk group was significantly lower than that of the low-risk group (*P* < 0.0001), indicating that the six miRNAs constituting the model were more predictive of the prognosis of the samples.

### Independent predictive capability of the model

**Figure 7:**
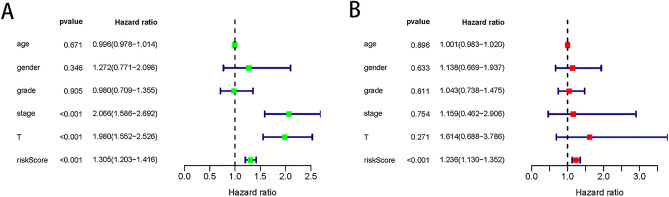
Independent predictive capability for prognostic model. Univariate (**A**) and multivariate (**B**) regression analyses of prognostic model and clinical indicators with overall survival. *P* values less than 0.05 were considered statistically significant.

The univariate results showed that stage, T-stage, and the prognostic model could have prognostic value. In the multivariate Cox regression model, only the prognostic model could be used as a prognostic-related independent predictor (Fig. 7).

### Differential expression of 6 miRNAs in T-stage, N-stage and M-stage

**Figure 8:**
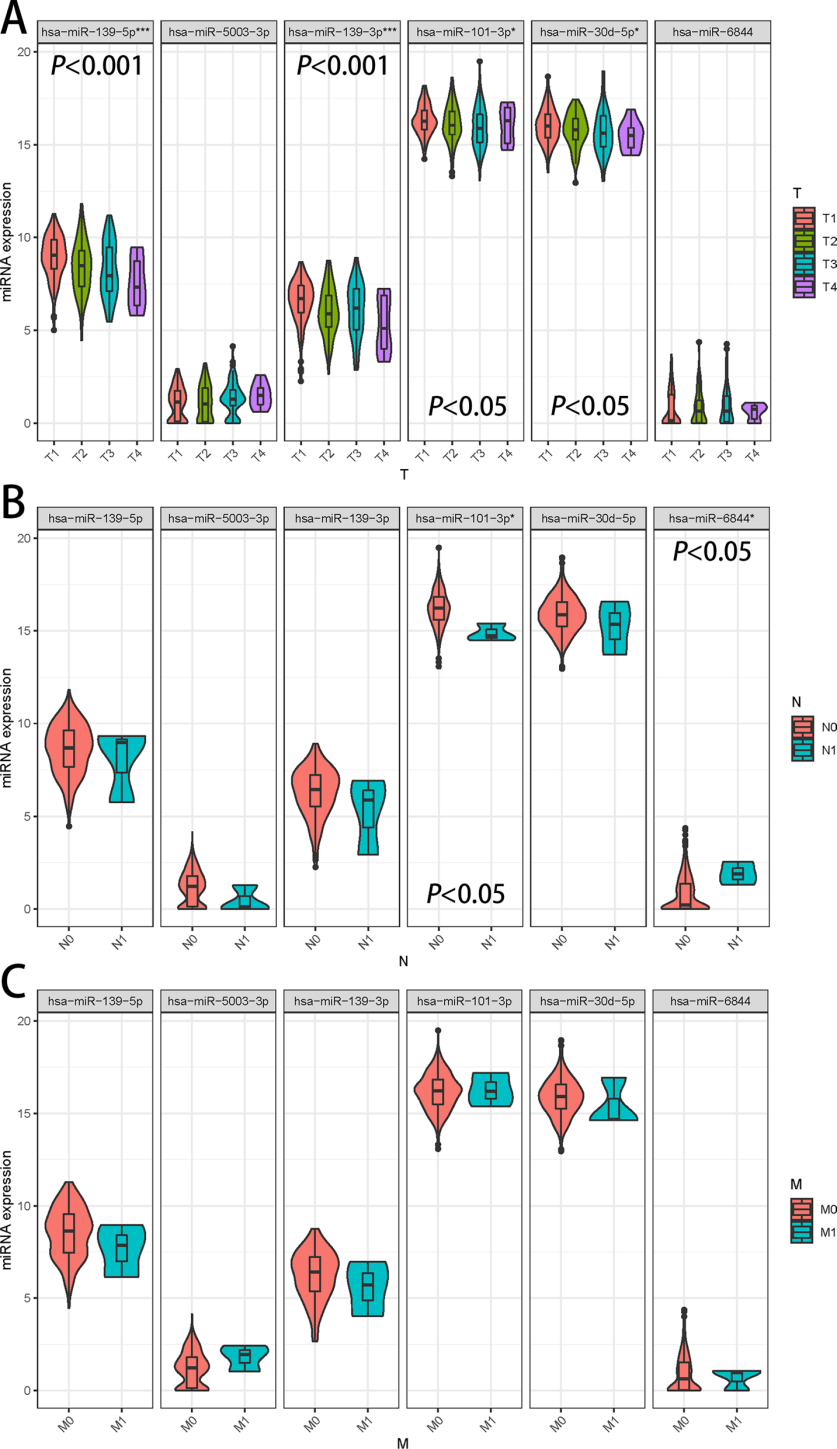
Analysis of 6 miRNAs in T-stage and N-stage. Expression analysis of 6 miRNAs in T-stage (**A**), N-stage (**B**) and M-stage (**C**). Different colors indicate different progressions. *P less than 0.05, ***P less than 0.001.

Violin plots (Fig. 8A–C) were used to examine the expression of the six miRNAs in T-stage and N-stage to hypothesize on the pathways in which the six miRNAs might play a role. The results showed that the expression of four miRNAs, including hsa-miR-139-3p, hsa-miR-139-5p, hsa-miR-101-3p, and hsa-miR-30d-5p, was significantly different at different T-stage. By comparing the mean values, it could be found that the expression of hsa-miR-139-3p, hsa-miR-139-5p and hsa-miR-30d-5p decreased gradually with the increase of tumor volume, and hsa-miR-101-3p decreased gradually from T1 to T3 phase and increased abruptly in T4 phase. These results suggested that these four miRNAs may function as regulators of tumor cell proliferation.

In N stage, the expression of hsa-miR-101-3p gradually decreased and that of hsa-miR-6844 gradually increased with the progression of N stage, suggesting that these two miRNAs may influence the lymph node metastasis of tumors and play a role in tumorigenesis development.

These six miRNAs showed no changes across M stages. However, the mean levels of these six miRNAs showed some variation.

### Prediction of target genes

**Figure 9:**
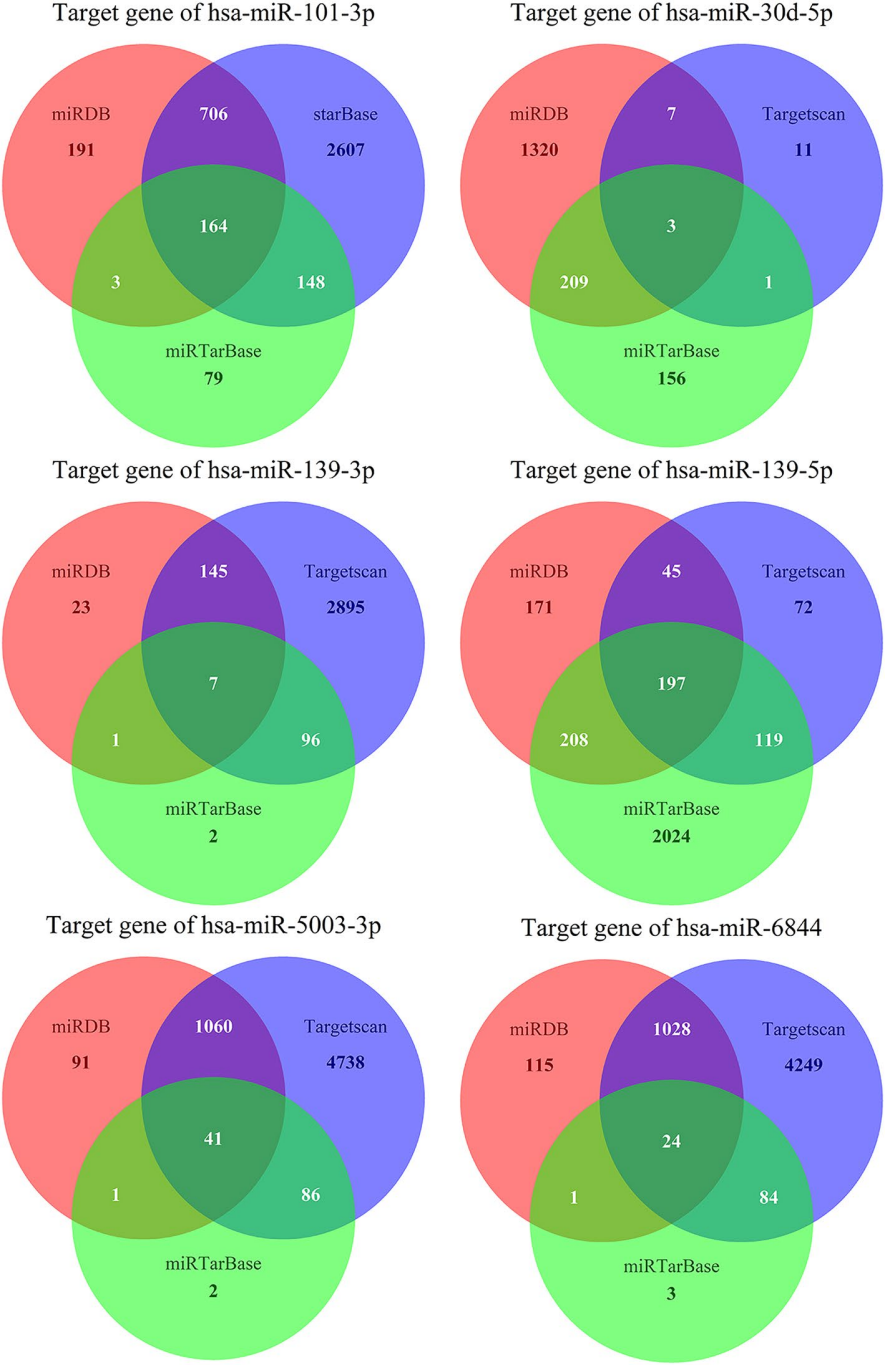
Prediction results of 6 miRNAs corresponding to mRNA. The three circles in red, green and blue indicate the predicted results in each of the three databases. The part where the three circles cross is the final result.

We predicted the mRNAs that would bind to DEmiRNAs and further investigated the functions of these mRNAs in humans. There were 436 corresponding mRNAs for the six miRNAs, including 164 for hsa-miR-101-3p, 3 for hsa-miR-30d-5p, 7 for hsa-miR-139-3p, 17 for hsa-miR-139-5p, 41 for has-miR-5003-3p, and 24 for hsa-miR-6844 (Fig. 9).

### Screening for hub genes

**Figure 10:**
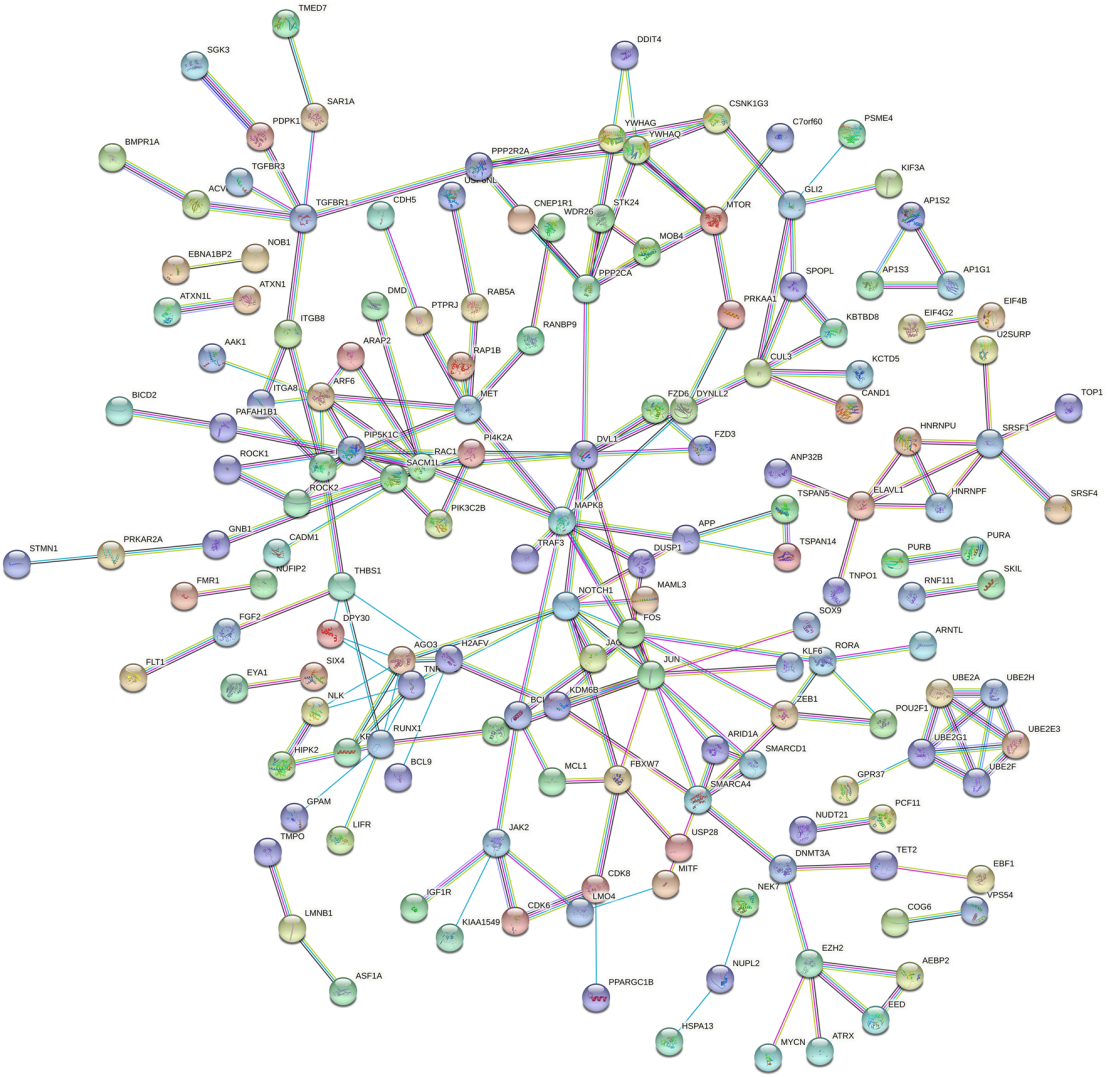
PPI plotted corresponding to mRNA. The circles represent the different mRNAs, and the richer the line indicates the higher the degree value.

**Figure 11:**
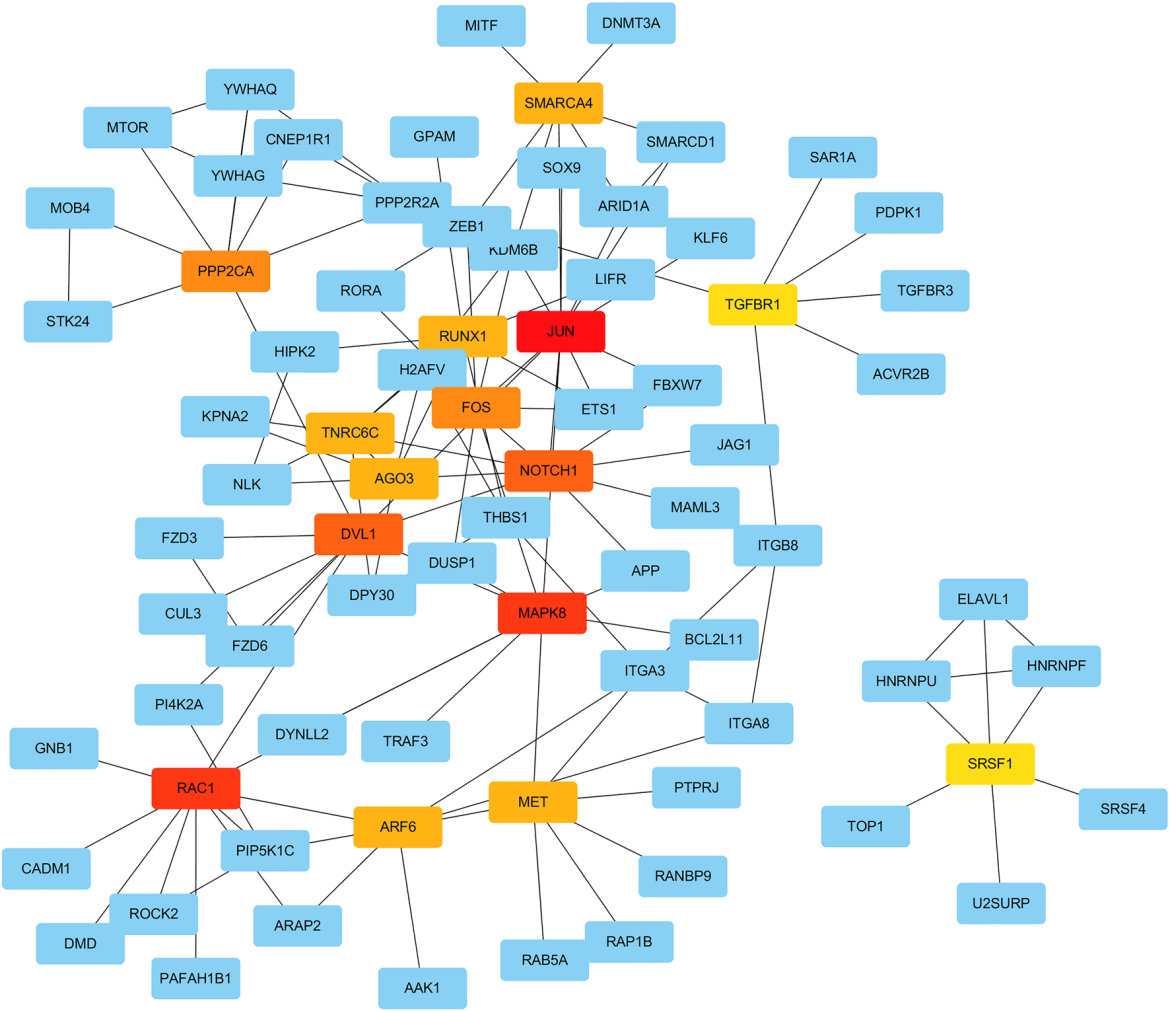
Hub genes based on Cytohubba calculations. A total of 15 hub genes were selected, with more red colors indicating more important genes in the reciprocal network.

**Figure 12:**
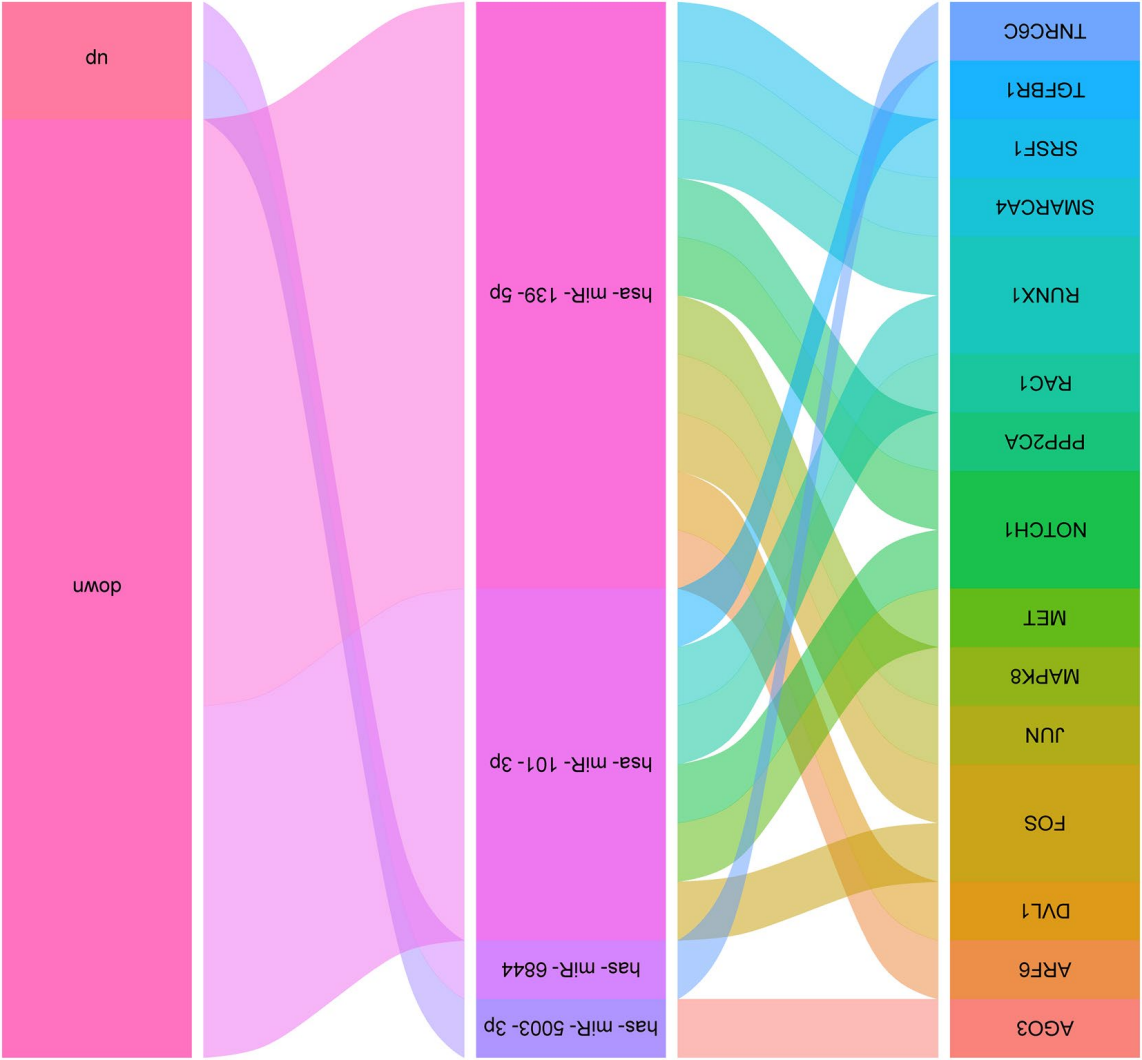
Sankey diagram of hub genes, miRNAs and up- and down-regulation relationships. Each rectangle represents a gene. The relationship between each gene and miRNA and miRNA up- and down-regulation is visualized based on the size of the rectangle and the relationship between the line segments.

We imported the target genes into the String database and perform PPI analysis to further confirm the hub genes in the targeted mRNA and speculate on their function. Sankey diagrams are used to show how hub genes and associated miRNAs are related. The result of the analysis in the String database is shown in Fig. 10. The results sorted by degree value from most to least showed that the top 15 hub genes were JUN, MAPK8, RAC1, NOTCH1, DVL1, PPP2CA, FOS, ARF6, AGO3, RUNX1, TNRC6C, MET, SMARCA4, SRSF1, and TGFBR1 (Fig. 11). The results of Sankey diagram (Fig. 12) showed that a total of four miRNAs were corresponding to hub genes, namely has-miR-5003-3p, hsa-miR-6844, hsa-miR-101-3p, hsa-miR-139-5p, of which has-miR-5003-3p and hsa-miR-6844 were overexpressed miRNAs, and hsa-miR-101-3p and hsa-miR-139-5p were down-regulated miRNAs.

### GO, KEGG analysis of hub mRNAs

**Figure 13:**
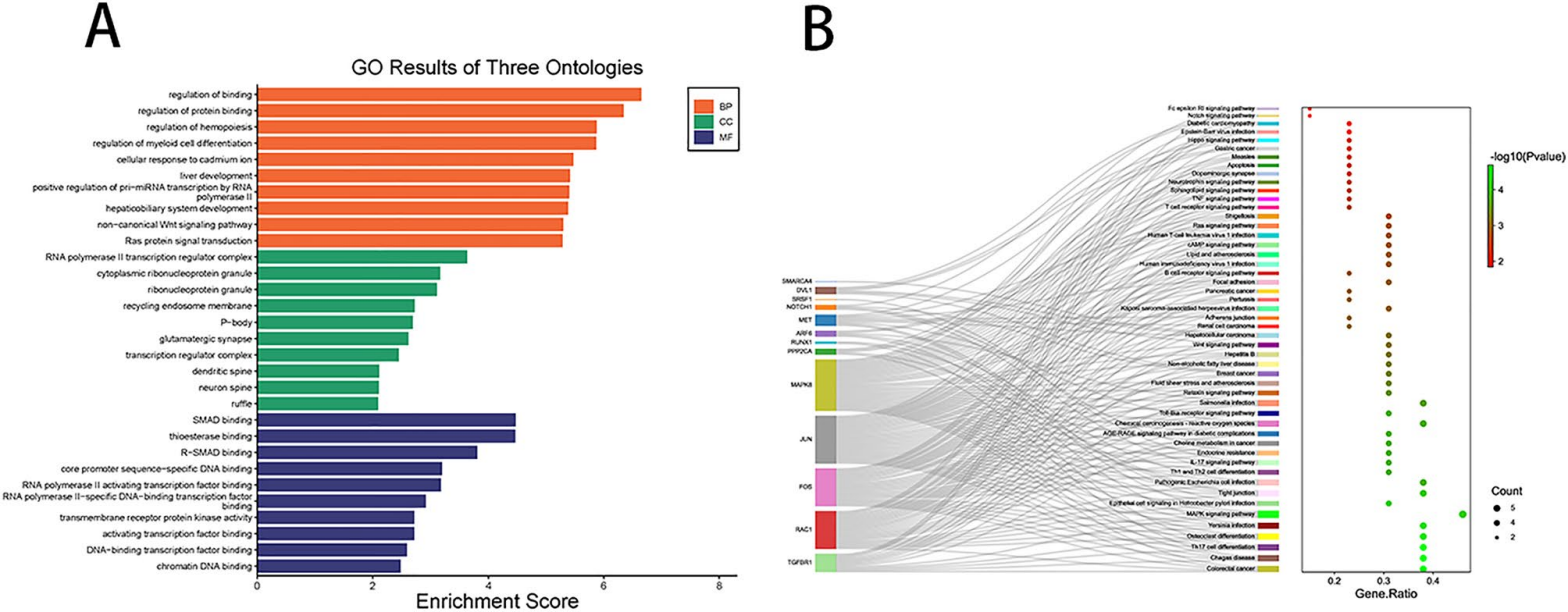
GO and KEGG analysis. GO analysis of hub mRNAs (**A**), horizontal coordinates indicate enrichment scores and vertical coordinates indicate the top ten enrichment results of hub mRNAs in Biological Process, Cellular Component and Molecular Function. The results of KEGG analysis (**B**), and the connecting lines in the diagram indicate the relationship between the hub gene and the pathway. The size of the bubbles in the right panel indicates count number, and the more red color indicates the smaller *p*-value.

GO (Table 1) and KEGG enrichment analysis of hub mRNAs showed that in biological processes, hub mRNAs were mainly enriched in protein binding, hematopoiesis, regulation of bone marrow cell differentiation, and development of liver and hepatobiliary system. In cellular components, hub mRNAs were mainly enriched in RNA polymerase II transcriptional regulator complex, cytoplasmic ribonucleoprotein granule. In molecular function, the hub mRNAs were mainly enriched in the binding of SMAD, thioesterase, and R-SMAD (Fig. 13A and Table 2). KEGG pathway analysis showed that the hub mRNAs were mainly concentrated in colorectal cancer, Th17 cell differentiation, and osteoblast differentiation (Fig. 13B).

**Table 1:** GO analysis of hub mRNAs.

ID	Description	Count	*p* value
Biological process			
GO:0051098	Regulation of binding	6	2.25E−07
GO:0043393	Regulation of protein binding	5	4.58E−07
GO:1903706	Regulation of hemopoiesis	6	1.34E−06
GO:0045637	Regulation of myeloid cell differentiation	5	1.36E−06
GO:0071276	cellular response to cadmium ion	3	3.37E−06
GO:0001889	Liver development	4	3.83E−06
GO:1902895	Positive regulation of pri-miRNA transcription by RNA polymerase II	3	3.95E−06
GO:0061008	Hepaticobiliary system development	4	4.16E−06
GO:0035567	Non-canonical Wnt signaling pathway	4	5.03E−06
GO:0007265	Ras protein signal transduction	5	5.2E−06
Cellular component			
GO:0090575	RNA polymerase II transcription regulator complex	3	0.000232
GO:0036464	Cytoplasmic ribonucleoprotein granule	3	0.000683
GO:0035770	Ribonucleoprotein granule	3	0.000772
GO:0055038	Recycling endosome membrane	2	0.001889
GO:0000932	P-body	2	0.002023
GO:0098978	Glutamatergic synapse	3	0.002406
GO:0005667	Transcription regulator complex	3	0.003521
GO:0043197	Dendritic spine	2	0.007742
GO:0044309	Neuron spine	2	0.007914
GO:0001726	Ruffle	2	0.008087
Molecular function			
GO:0046332	SMAD binding	3	3.37E−05
GO:0031996	thioesterase binding	2	3.42E−05
GO:0070412	R-SMAD binding	2	0.000156
GO:0001046	Core promoter sequence-specific DNA binding	2	0.000632
GO:0001102	RNA polymerase II activating transcription factor binding	2	0.00066
GO:0061629	RNA polymerase II-specific DNA-binding transcription factor binding	3	0.001217
GO:0019199	Transmembrane receptor protein kinase activity	2	0.001899
GO:0033613	Activating transcription factor binding	2	0.001899
GO:0140297	DNA-binding transcription factor binding	3	0.002575
GO:0031490	Chromatin DNA binding	2	0.003304

**Table 2:** KEGG analysis of hub mRNAs.

ID	Description	*p* value	Count
hsa05210	Colorectal cancer	1.43E−07	5
hsa05142	Chagas disease	3.38E−07	5
hsa04659	Th17 cell differentiation	4.5E−07	5
hsa04380	Osteoclast differentiation	1.05E−06	5
hsa05135	Yersinia infection	1.47E−06	5
hsa04010	MAPK signaling pathway	2.98E−06	6
hsa05120	Epithelial cell signaling in Helicobacter pylori infection	3.42E−06	4
hsa04530	Tight junction	4.15E−06	5
hsa05130	Pathogenic Escherichia coli infection	8.8E−06	5
hsa04658	Th1 and Th2 cell differentiation	1.02E−05	4

## Discussion

HCC is one of the most deadly malignant digestive cancers worldwide, with high morbidity and mortality^22^. In recent years, the incidence of HCC has been on the rise due to environmental factors, immunizations, and changes in people’s lifestyles^23^. However, almost 60–80% of patients with HCC are diagnosed at an advanced stage and therefore deprived of surgical treatment, with a 5-year survival rate < 12.5%^24,25^. Although there are clinical staging systems such as Tumor Node Metastasis (TNM) staging, Barcelona Clinic Liver Cancer (BCLC) staging, Cancer of the Liver Italian Program (CLIP) scoring to predict the prognosis of patients, these systems have limited ability and cannot better enable clinicians to stratify management to develop personalized treatment plans. Therefore, it is important to identify reliable and valid prognostic biomarkers for HCC.

MiRNAs are widely available in the human body and can be detected in peripheral blood, and therefore have considerable advantages in terms of clinical applications. In this study, we downloaded 372 HCC samples and 50 adjacent normal tissue samples of HCC patients from TCGA and identified six prognosis-related miRNAs using bioinformatics approach. The prognostic model was constructed and validated using six prognosis-related miRNAs, including hsa-miR-139-3p, hsa-miR-139-5p, hsa-miR-101-3p, and hsa-miR-30d-5p, hsa-miR-5003-3p, and hsa-miR-6844. Compared with the AFP model^26^, our model has a better predictive ability. The model has AUC values above 0.7 at 1, 3 and 5 years in both the training and validation sets. Many studies have identified hub mRNAs that play key role in tumor progression and established goodprognostic models^27^. Compared with mRNA, miRNAs have the advatages of structural stabilityand strong cancer type-specific expression. Our model also performs better in the validation set incomparison to published mRNA prediction models^28^. In comparison to other miRNA-based prognostic models^29,30^, the described models frequently only assess the 3-year or 5-year C-index, which is insufficient. And our model is stable after 1, 3, and 5 years, with a C index greater than 0.7. Meanwhile, the calibration curves of the model were also plotted in this study, showing that the prediction results of the model in 1, 3, and 5 years overlap more closely with the actual results, further validating the predictive ability of the model. In terms of model utilization, compared to the study by Su et al.^31^, we improved the applicability of the model by visualizing the model by drawing nomograms and grouping patients by risk scores. When used by clinicians, the prognosis of patients can be predicted visually and precise treatment can be implemented. In addition, we evaluated the independent predictive ability of the model. The results showed that the model had independent predictive ability and could be used to test the prognosis of patients independently of other clinical factors. The prediction role of miRNA in tumor prognosis has been investigated in several studies^32,33^. However, in general, our miRNA prognosis model may be used directly via nomogram, and the C index after 5 years is greater than 0.7, indicating superior and more stable prognostic capacity.

There is growing evidence that has-miR-139-5p, hsa-miR-101-3p, and has-miR-30d-5p affect cellular functions and play a part in the emergence and spread of several cancer types^34–38^. In HCC, in agreement with previous reports^39–43^, our results showed that downregulation of hsa-miR-139-5p, hsa-miR-101-3p, and has-miR-30d-5p was significantly associated with poor survival in HCC patients. The levels of these three miRNAs gradually decreased with the aggravation of T-stage in HCC patients. The expression level of hsa-miR-101-3p gradually decreased in the N stage. This suggests that hsa-miR-139-5p and hsa-miR-30d-5p may influence the prognosis of patients by affecting the proliferation of tumor cells, and hsa-miR-101-3p influences the disease process of HCC patients by affecting both the proliferation and migration of HCC cells. Therefore, these three miRNAs may be effective targets for the treatment of HCC.

Currently, there are relatively few studies on hsa-miR-6844 and hsa-miR-5003-3p in HCC patients. Elevated expression levels of hsa-miR-6844 and hsa-miR-5003-3p have been found in breast and cervical cancers, respectively^44,45^. Similarly, our study found that the expression of these two miRNAs was significantly elevated in the tumor tissues of HCC patients, and the increased expression levels were associated with a poor prognosis. At the TNM stage, the expression level of hsa-miR-6844 increased with the progression of N stage, implying that this miRNA may influence patient prognosis by affecting cell migration. In addition, hsa-miR-5003-3p expression levels failed to demonstrate statistically significant results. In future studies, increasing the sample size may allow significant differences to occur.

In recent years, many studies have reported the role of hsa-miR-139-3p in cancer. Our study found that this miRNA was highly expressed in HCC patient tissues compared to normal tissues, and the high expression was accompanied by a poor prognosis. In contrast to our findings, Qin et al.^46^ found that low expression of hsa-miR-139-3p was associated with a poor prognosis. Further studies are needed in the future to explore the relationship. In our study hsa-miR-139-3p showed differences in T-stage of HCC patients, suggesting that this miRNA acts mainly by affecting cell proliferation, which is similar to the results of other studies^47^.

Most miRNAs exhibit strong correlations. To avoid such problems, we performed univariate and multivariate Cox regression analyses, followed by a LASSO analysis to screen hub miRNAs in the current study. When compared to a stepwise regression, LASSO regression can keep variables that have impacts on the dependent variable that are both significant and non-significant, reducing the estimation deviation^48^. In actuality, prognostic modeling of HCC patients in current research heavily relies on LASSO, one of the developing research methodologies. Liang et al.^49^ successfully built prognostic-related models by using LASSO to search patients for OS-related ferroptosis-related genes. Using LASSO, Yang et al.^50^ searched gene models relevant to macrophages and mapped the nomogram. Thus, it is obvious that LASSO has special benefits for research involving genes. Similar to the previous studies, we used LASSO to screen prognosis-related miRNAs, which increased the accuracy of the model.

Another advantage of this paper is to predict of the targeted mRNAs of miRNA. Using the PPI protein interaction network analysis of target genes, we finally screened out the 15 most important mRNAs and visualized their relationships with corresponding miRNAs by ranking them from highest to lowest degree values. The 15 mRNAs are: JUN, MAPK8, RAC1, NOTCH1, DVL1, PPP2CA, FOS, ARF6, AGO3, RUNX1, TNRC6C, MET, SMARCA4, SRSF1, TGFBR1. The relationship between most of hub mRNAs and HCC has been reported by previous studies. The roles of mRNAs such as JUN^51^, MAPK8^45^ and FOS^52^ in HCC, for example, have been experimentally validated. Furthermore, studies on SMARCA4, SRSF1, and TNRC6C mainly focused on breast cancer^53^, cervical cancer^54^, and thyroid cancer^55,56^, and their roles in HCC have been less studied. The prognosis of HCC patients may also be influenced by these less-researched mRNAs.

However, this study has some limitations. The study sample is primarily white and black, which may mean that other ethnic groups are underrepresented, among other drawbacks. Additionally, we solely used data from the TCGA without any outside validation because there were generally not enough clinical data in the Gene Expression Omnibus (GEO) dataset, which may have caused some partial bias in the results. Cohort studies will be used to validate the model in later research, and more accurate statistical techniques will be used to improve model accuracy.

## Conclusions

In conclusion, we developed a regression model utilizing miRNAs to predict the prognosis of HCC patients over 5-years with high model sensitivity and strong predictive capacity. Moreover, we enhanced the practical usability of the model by building a nomogram. We also built a network of miRNAs and mRNAs and investigated the role of targeting mRNAs.

## Data Availability

The datasets used and/or analysed during the current study are available from the cancer genome database (TCGA) https://portal.gdc.cancer.gov.

## Abbreviations

HCC: Hepatocelluar carcinoma
miRNA: MicroRNA
DEmiRNAs: Differentially expressed miRNAs
DCA: Decision curve analysis
OS: Overall survival
GO: Gene Ontology
KEGG: Kyoto encyclopedia of genes and genomes
TCGA: The cancer genome database
PPI: Protein–Protein interaction
ROC: Receiver operating characteristic
TNM: Tumor node metastasis
BCLC: Barcelona Clinic Liver Cancer
CLIP: Cancer of the Liver Italian Program
GEO: Gene expression omnibus
